# African Baobabs with False Inner Cavities: The Radiocarbon Investigation of the Lebombo Eco Trail Baobab

**DOI:** 10.1371/journal.pone.0117193

**Published:** 2015-01-26

**Authors:** Adrian Patrut, Stephan Woodborne, Karl F. von Reden, Grant Hall, Michele Hofmeyr, Daniel A. Lowy, Roxana T. Patrut

**Affiliations:** 1 Faculty of Chemistry, Dept. of Chemistry, Babeş-Bolyai University, Arany Janos 11, Cluj-Napoca, Romania; 2 iThemba Laboratories, Box 722, Somerset West, South Africa; 3 NOSAMS Facility, Dept. of Geology & Geophysics, Woods Hole Oceanographic Institution, Woods Hole, Massachusetts, United States of America; 4 Mammal Research Institute, University of Pretoria, Pretoria, South Africa; 5 SANParks Scientific Services, Pvt Bag x402, Skukuza, South Africa; 6 Nova University, 5000 Dawes Ave., Alexandria, Virginia, United States of America; 7 Faculty of Biology and Geology, Babeş-Bolyai University, Gh. Bilascu 44, Cluj-Napoca, Romania; Henan Agricultural Univerisity, CHINA

## Abstract

The article reports the radiocarbon investigation results of the Lebombo Eco Trail tree, a representative African baobab from Mozambique. Several wood samples collected from the large inner cavity and from the outer part of the tree were investigated by AMS radiocarbon dating. According to dating results, the age values of all samples increase from the sampling point with the distance into the wood. For samples collected from the cavity walls, the increase of age values with the distance into the wood (up to a point of maximum age) represents a major anomaly. The only realistic explanation for this anomaly is that such inner cavities are, in fact, natural empty spaces between several fused stems disposed in a ring-shaped structure. We named them false cavities. Several important differences between normal cavities and false cavities are presented. Eventually, we dated other African baobabs with false inner cavities. We found that this new architecture enables baobabs to reach large sizes and old ages. The radiocarbon date of the oldest sample was 1425 ± 24 BP, which corresponds to a calibrated age of 1355 ± 15 yr. The dating results also show that the Lebombo baobab consists of five fused stems, with ages between 900 and 1400 years; these five stems build the complete ring. The ring and the false cavity closed 800–900 years ago. The results also indicate that the stems stopped growing toward the false cavity over the past 500 years.

## Introduction

The African baobab (*Adansonia digitata* L.), a tropical angiosperm which belongs to the Bombacoideae, a subfamily of Malvaceae, is the best-known and the largest of the nine *Adansonia* species [1–4]. The African baobab has a natural distribution in the tropical arid savanna regions of mainland Africa between the latitudes 16° N and 26° S. It can also be found in several African islands and outside Africa, in different tropical areas, where it has been introduced [1,2,5–7].

The familiar picture of the African baobab is that of an almost grotesque tree, with a girth out of proportion to its height. The very large size of several individuals suggests that this iconic tree of the African landscape lives to an old age and might be the longest living angiosperm [8–13]. However, determining the age of large baobabs has proven to be a very difficult task.

The faint growth rings produced by the African baobab cannot be used even for dating fallen large and old trees. An accurate ring count is not possible, as growth rings may no longer be produced and observed in certain areas of the trunk of old baobabs and also because of the presence of large internal hollow parts. Therefore, the sole accurate method for determining the age of African baobabs is considered to be radiocarbon dating [13–18].

In 2005, we started an in-depth research aimed to elucidate several controversial or poorly understood aspects regarding the architecture, growth and age of the African baobab. This research is based on our new approach which also allows to investigate and date standing and live specimens. Our approach consists of AMS radiocarbon dating of small wood samples collected especially from inner cavities, but also from deep incisions/entrances in the stems, fractured/broken stems and from the outer part/exterior of large baobabs [16,19].

Many baobabs, especially old specimens, have large hollow parts, mainly in the central area of their trunk/stems [2,20]. The large normal cavities are formed by wood removal (as a result of fungi decay, fire, animal damage, human activity) and the pith/centre of the stem is located inside the cavity. Therefore, for samples collected from normal cavities, the age values should decrease continuously from the cavity walls toward the outer part of the stem [19,21].

In most cases we found, however, that the age values exhibit a continuous increase from the cavity walls up to a certain distance into the wood, after which they decrease toward the outer part. The only explanation for this anomaly, which represents a reproducible experimental finding, is that such inner cavities are in fact natural empty spaces between fused stems disposed in a closed ring-shaped structure. We named them false cavities. The oldest part of the fused stems is located between the false cavity walls and the outer part/exterior of each stem, always closer to the cavity.

Here we present the AMS radiocarbon investigation results of the Lebombo Eco Trail baobab, the first specimen for which we discovered the presence of false cavities.

## Materials and Methods

### Ethics statement

The investigation and collection of small samples from the Lebombo Eco Trail baobab was approved and authorised by The South African National Parks and The Kruger National Park. The Kruger National Park also provided scientific assistance and armed escort for the on-site investigation. The baobab was not endangered in any way by the sampling. After each coring, the increment borer was disinfected with methyl alcohol. The small coring holes were sealed with Steriseal (Efekto), a special polymer sealing product, for preventing any possible infection of the tree.

A more invasive sampling in areas of the so-called fusion or separation between stems in monumental baobabs, for revealing the internal structure, will never be allowed by the authorities.

Two figures include images of persons participating in the on-site investigation. These individuals, who are also co-authors of the paper, have given written informed consent (as outlined in PLOS consent form) to publish their images as case details.

### The Lebombo Eco Trail baobab and its area

The Lebombo baobab/tree is located slightly inside Mozambique, between the Kruger National Park and the Limpopo Transfrontalier Park, at only 10.5 m from the current border with South Africa. The Limpopo Eco Trail 4x4 of the Kruger National Park, the only accessible road to the tree, is closed to tourists. Its GPS coordinates are 23°15.765′ S, 031°33.309′ E and the altitude is 290 m. Mean annual rainfall in the area is 438 mm (Shingwedzi station).

**Fig 1:**
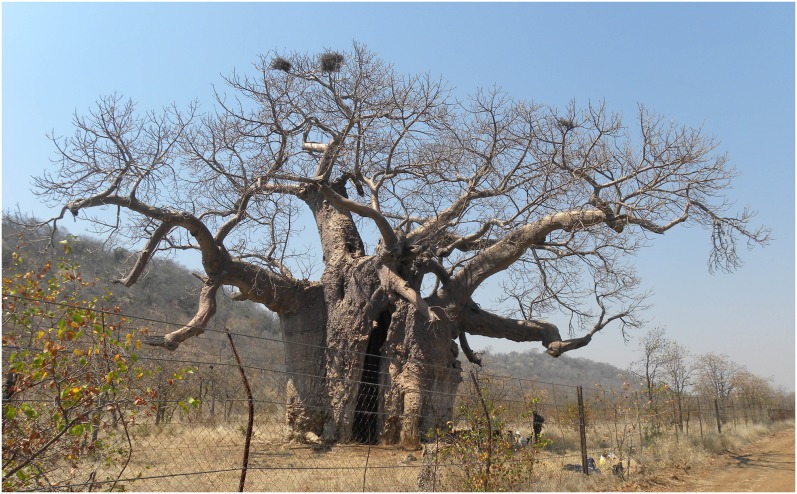
General view of the Lebombo baobab, showing also the high entrance to the cavity. The photograph is taken from the Eco Trail 4x4 of the Kruger National Park. One can observe the fence which marks the border between South Africa and Mozambique. (The photograph was taken by Roxana Patrut).

**Fig 2:**
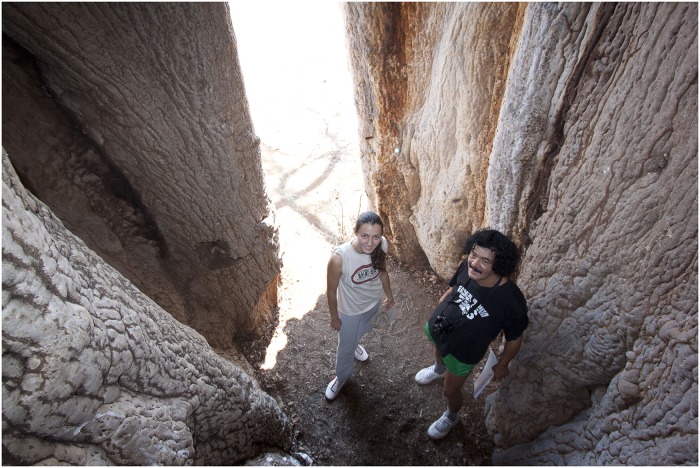
Top view of the tall false inner cavity of Lebombo baobab, which is defined by five fused stems. One can notice the ellipsoidal shape of the cavity base and the bark which covers the cavity walls. The false cavity has a large opening toward the outside. (The photograph was taken by Stephan Woodborne).

The tree has a peculiar look, with long branches which resemble to mammoth tusks. It has a maximum height of 18.5 m, the circumference at breast height (cbh; at 1.30 m above ground level) is 21.44 m and the overall volume (trunk and branches) is around 220 m^3^ (Fig. 1). The big trunk has a central cavity, with an ellipsoidal basis (with the axes of 2.72 x 2.20 m at ground level and 2.92 x 2.22 m at breast height), a height of 7.80 m and a very high opening toward the exterior (6.49 m). The inner cavity walls are completely covered by bark (Fig. 2).

### Sample collection

**Fig 3:**
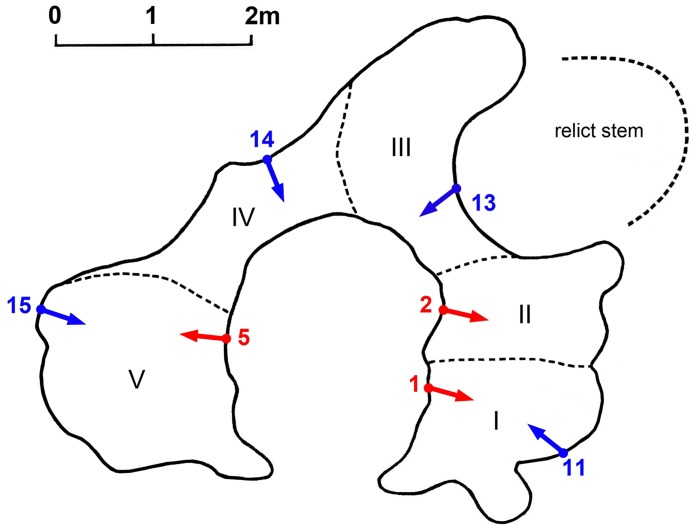
Cross-section of the Lebombo baobab (at 1.30 m above ground), showing the false cavity, the projection of the positions of the three internal sampling points (labelled 1, 2 and 5) and of the four external sampling points (labelled 11, 13, 14 and 15), with the sampling directions. The five fused stems (labelled I, II, III, IV and V) are also displayed.

A number of seven samples were collected as follow: three samples (labelled 1, 2 and 5) were collected from the inner cavity walls and other four samples (labelled 11, 13, 14 and 15) were extracted from the outer part/exterior of the trunk. The sampling positions are shown in Fig. 3.

The samples were collected using a Haglöf CH 600 increment borer (60 cm long, 0.54 cm inner diameter). A number of 19 small pieces/segments of the length of 0.1 cm were extracted from determined positions of the original seven samples. These segments (labelled a, b, c, d or e) were processed and investigated by AMS radiocarbon dating.

### Sample preparation

The standard acid-base-acid pretreatment method [22] was used for removing soluble and mobile organic components. The resulting samples were combusted to CO_2_, via the closed tube combustion method [23]. Then, CO_2_ was reduced to graphite on iron catalyst, under hydrogen atmosphere [24]. Eventually, the resulting graphite samples were analysed by AMS.

*AMS measurements*. AMS radiocarbon measurements were performed at the NOSAMS Facility of the Woods Hole Oceanographic Institution by using the Pelletron Tandem 500 kV AMS system [25,26]. The obtained fraction modern values, corrected for isotopic fractionation with the normalized δ^13^C value of-25^0^/_00_, were ultimately converted to a radiocarbon date.

### Calibration

Fraction modern values were calibrated and converted into calendar ages with the OxCal v4.2 for Windows [27], by using the SHCal13 atmospheric data set [28].

## Results and Discussion

### Wood samples

The main sampling data are displayed in Table 1. The sampling positions and directions were chosen at convenient heights (between 1.30–1.56 m), according to the stems profile/relief in the selected area, in order to assure an optimal penetration of the increment borer. In one case (sample 14), however, the sampling point was higher (2.25 m), given the very irregular profile of the stem at lower heights. Even if, except for samples 13 and 14, the penetration of the borer in the wood was between 0.50–0.60 m, the lengths of the collected samples are very different. All three samples collected from the cavity (1, 2 and 5) consist of two pieces, due to the existence of one hollow part along the sample; the length of the hollow part, *i*.*e*., the missing wood, is marked in Table 1 as x, y, z. On the other hand, the deepest end of the three inner samples corresponds to the beginning of a larger hollow part around the point of maximum age in the sampling direction. The four samples collected from the exterior/outer part (11, 13, 14 and 15), which are considerably younger, consist each of only one piece. In the case of two external samples (13 and 14), the penetration of the borer was more difficult and shorter in the regrowth layers triggered by the collapse of an adjacent stem. One should mention that the inner samples 1 and 5 are quasi-opposite to the external samples 11 and 15, *i*.*e*., they have been collected from the same stem in opposite directions.

**Table 1:** Sampling data.

Sample code	Sample length (10^–2^ m)	Sampling height (m)	Width of cavity wall (m)
1	14.1+x+16.0 = 30.1+x	1.42	1.80
2	17.2+y+18.9 = 36.1+y	1.56	1.70
5	13.8+z+9.0 = 22.8+z	1.53	2.05
11	46.0	1.43	1.80
13	15.0	1.30	0.80
14	22.2	2.25	0.85
15	39.1	1.33	2.05

### AMS results and calibrated ages

Fraction modern values and radiocarbon dates of the seven samples are listed in Tables 2 and 3. Radiocarbon dates and errors were rounded to the nearest year.

**Table 2:** AMS Radiocarbon dating results and calibrated calendar ages of samples collected from the cavity of the Lebombo Eco Trail baobab.

Sample (Segment)	Depth^1^ (10^–2^ m)	Fraction modern [error]	Radiocarbon date [error] (^14^C yr bp)	Cal ad range 1-σ [confidence interval]	Sample age [error] (cal yr)	NOSAMS Accession #
LEB-1(a)	0.5	0.9521 [±0.0031]	394 [± 25]	**1462–1509 [35.7%]** 1580–1620 [32.5%]	530 [± 25]	OS-83728
LEB-1(b)	7.5	0.8996 [±0.0028]	850 [± 22]	1216–1266 [68.2%]	775 [± 25]	OS-84375
LEB-1(c)	16 + x	0.8861 [±0.0032]	971 [± 26]	**1045–1093 [37.6%]** 1107–1122 [10.0%] 1128–1156 [20.6%]	945 [± 25]	OS-84388
LEB-1(d)	29+x	0.8782 [±0.0030]	1043 [± 24]	**995–1045 [51.2%]** 1090–1108 [12.1%] 1121–1128 [5.0%]	995 [± 25]	OS-85870
LEB-2(a)	0.5	0.9513 [±0.0030]	401 [± 24]	**1461–1506 [41.2%]** 1588–1617 [27.0%]	530 [± 20]	OS-87452
LEB-2(b)	16	0.9180 [±0.0030]	687 [± 24]	1300–1320 [26.1%] **1350–1386 [42.1%]**	650 [± 20]	OS-83727
LEB-2(c)	20+y	0.8870 [±0.0030]	963 [± 24]	**1046–1090 [36.9%]** 1108–1120 [7.1%] 1130–1162 [24.3%]	945 [± 20]	OS-86092
LEB-2(d)	35+y	0.8374 [±0.0030]	1425 [± 24]	642–672 [68.2%]	1355 [± 15]	OS-92686
LEB-5(a)	1	0.9576 [±0.0030]	348 [± 24]	**1509–1580 [59.9%**] 1620–1630 [8.3%]	470 [± 35]	OS-87449
LEB-5(b)	12	0.9169 [±0.0030]	697 [± 24]	1296–1318 [28.7%] **1353–1384 [39.5%]**	645 [± 15]	OS-87447
LEB-5(c)	16+z	0.9015 [±0.0031]	833 [± 25]	1226–1266 [68.2%]	770 [± 20]	OS-90734
LEB-5(d)	22+z	0.8908 [±0.0029]	929 [± 23]	1150–1209 [68.2%]	835 [± 30]	OS-90740

^1^ Depth in the wood from the sampling point.

**Table 3:** AMS radiocarbon dating results and calibrated calendar ages of samples collected from the exterior of the Lebombo Eco Trail baobab.

Sample (Segment)	Depth^1^ (10^–2^ m)	Fraction modern [error]	Radiocarbon date [error] (^14^C yr bp)	Cal ad range 1-σ [confidence interval]	Sample age [error] (cal yr)	NOSAMS Accession #
LEB-11(a)	22	0.9728 [±0.0037]	222 [± 30]	1664–1678 [10.5%] **1734–1800 [57.7%]**	245 [± 35]	OS-84389
LEB-11(b)	45.5	0.9473 [±0.0038]	435 [± 31]	**1450–1498 [60.6%]** 1599–1608 [7.6%]	540 [± 25]	OS-87450
LEB-13(a)	5	0.9979 [±0.0031]	17 [± 25]	**1816–1828 [42.9%]** 1894–1906 [25.3%**]**	190 [± 5]	OS-87454
LEB-13(b)	14.5	0.9919 [±0.0025]	65 [± 20]	**1816–1828 [35.0%]** 1894–1909[33.2%]	190 [± 5]	OS-86100
LEB-14(a)	20	0.9907 [±0.0032]	75 [± 26]	1816–1832 [24.6%] **1892–1922 [43.6%]**	105 [± 15]	OS-87711
LEB-15(a)	23	0.9704 [±0.0032]	241 [± 26]	1655–1672 [20.7%] **1743–1770 [25.4%]** 1779–1796 [22.1%]	260 [± 15]	OS-84390
LEB-15(b)	39	0.9606 [±0.0030]	323 [± 24]	**1512–1549 [33.8%]** 1561–1571 [6.7%] 1622–1648 [27.7%]	485 [± 20]	OS-89541

^1^ Depth in the wood from the sampling point.

Calibrated (cal) ages are also displayed in Tables 2 and 3. The 1-σ probability distribution was selected to derive calibrated age ranges. For four sample segments, the 1-σ distribution is consistent with only one range of calendar years, while for other 15 segments, the 1-σ distribution corresponds to two or three ranges of calendar years. For these segments, the confidence interval of one range is, with three exceptions, considerably greater than that of the others; therefore, it was selected as the cal ad range of the sample for the purpose of this discussion.

For obtaining single calendar age values of sample segments, we derived a mean calendar age of each segment from the selected range (marked in bold). Calendar ages of segments represent the difference between ad 2014 and the mean value of the selected range, with the corresponding error. Calendar ages and errors were rounded to the nearest 5 years.

The accession numbers are included in the last column of Tables 2 and 3.

### Dating results of samples collected from the cavity

**Fig 4:**
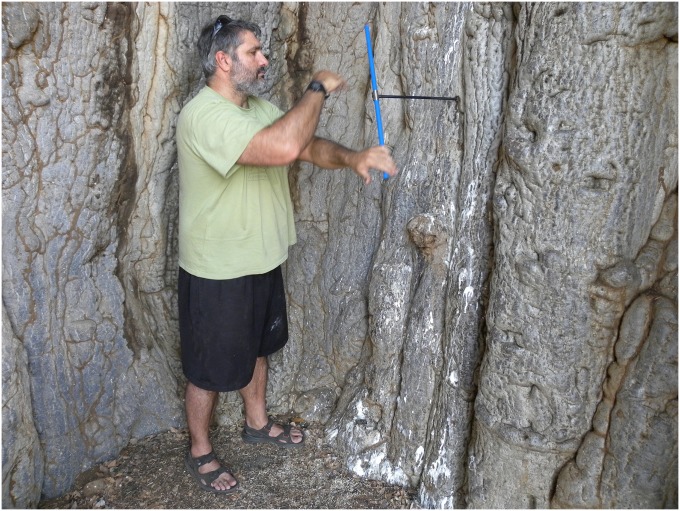
Collecting the oldest sample (Nr. 2) from the false cavity of the Lebombo baobab. (The photograph was taken by Adrian Patrut).

The dating results demonstrate that for all seven samples collected from the cavity or from the exterior, the age values show a continuous increase with the distance/depth into the wood. The most interesting are certainly the three samples collected from the cavity, *i*.*e*., 1, 2 and 5 (Fig. 4); from each of these samples we extracted and dated four segments, see Table 2. The age sequences along the samples indicate that the length of the hollow parts/missing wood, marked x, y and z, is of only several centimetres.

The maximum age values were recorded at the sample ends, *i*.*e*., the deepest segments are also the oldest. The oldest dated segment 2(d) originate from sample 2; its radiocarbon date was found to be 1425 ± 24 bp, which corresponds to a calibrated age of 1355 ± 15 yr. The ages of the oldest segments extracted from samples 1 and 5, namely 1(d) and 5 (d), have lower values; their radiocarbon dates of 1043 ± 24 bp and 929 ± 23 bp correspond to calibrated ages of 995 ± 25 and 835 ± 30 yr. As already mentioned, the total length of the cavity samples, including the estimated missing wood, suggests that the deepest end of each inner sample corresponds to another hollow part, which is located around the point of maximum age in the sampling direction. The very different old age values of the three cavity samples demonstrate that they originate from three different stems, which were labelled I, II and V, see Fig. 3.

Up to present, the normal/common cavities, which occur by wood removal, are the only ones mentioned in the literature for all tree species. In the case of quasi-central normal cavities, the age values of the cavity samples decrease continuously with the distance into the wood. The increase of age values of samples collected from the cavity walls with the distance into the wood (up to the point of maximum age) was the first major anomaly found by us in the radiocarbon investigation of the Lebombo baobab. The only possible explanation for this finding is that such cavities, which we named false cavities, are in fact natural empty spaces (which were never filled with wood) between several fused stems disposed in a closed ring-shaped structure.

The second significant dating anomaly was that the innermost rings of the cavity samples, which are adjacent to the cavity bark, were found to be several hundred years old instead of being very young. The radiocarbon ages of the innermost segments of the three cavity samples, *i*.*e*., 1(a), 2(a) and 5(a), which correspond to a distance of only 0.5 cm into the wood, are 394 ± 25 bp, 401 ± 24 bp and 348 ± 24 bp; these values correspond to calibrated ages of 530 ± 25, 530 ± 20 and 470 ± 35 yr. These anomalous results indicate that the stems of Lebombo baobab basically have stopped growing completely toward the false cavity over the past 470–530 yr, *i*.*e*., the growth stop started in the period ad 1485–1545. We consider that this growth stop was necessary for maintaining a stable internal architecture and preventing the collapse of the cavity and of the entire ring.

### Dating results of samples collected from the exterior/outer part

A number of two segments were extracted and dated from each of the external samples 11, 13 and 15, while from the shortest sample 14 only one segment was extracted. The obtained results are shown in Table 3. In the case of the longest external samples 11 and 15, which are opposite to the internal samples 1 and 5, the age values increase, as expected, with the distance into the wood. For sample 11, the calibrated ages of the two dated segments are 245 ± 35 yr (at 0.20 m) and 545 ± 20 yr (at 0.455 m). According to these values, the linear growth of the corresponding stem I toward the exterior was relatively slow and almost constant over the past 545 yr. Thus, the mean radial increase for the dated segments varied in this time frame in a narrow range, between 0.82–0.85 x 10^–3^ m yr^-1^. For sample 15, the calibrated ages of the two segments are 260 ± 15 yr (at 0.19 m) and 485 ± 20 yr (at 0.38 m); these values also show a slow decrease of the mean radial increase of stem V over the past 485 yr, from 0.84 x 10^–3^ to 0.73 x 10^–3^ m yr^-1^.

**Fig 5:**
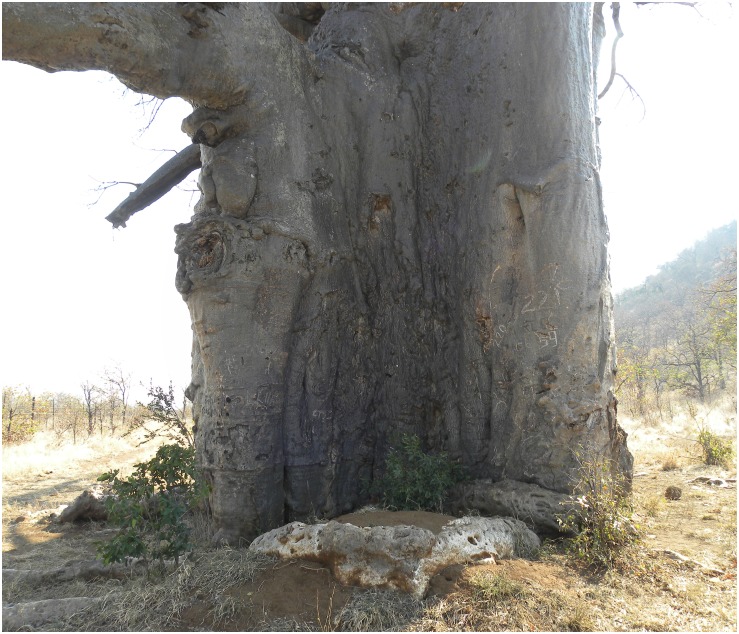
The image shows the relict stem in front of stem III. The pronounced convex shape of stem III indicates that it had to conform to the shape of the older relict stem. (The photograph was taken by Adrian Patrut).

**Fig 6:**
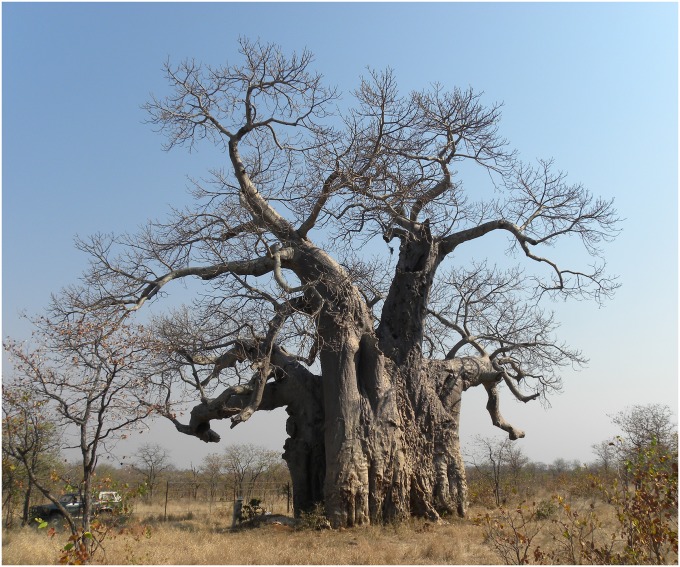
The canopy shape (without any branches toward the south-west) and the very irregular profile of stem IV (in front) suggest that another stem must have collapsed in its vicinity. (The photograph was taken by Adrian Patrut).

The segments extracted from the two short external samples 13 and 14 exhibit radiocarbon dates between only 17 ± 25 bp and 77 ± 22 bp. The calibration of such very low radiocarbon dates is very difficult and also uncertain, particularly with the southern data sets. In our case, the most probable calibrated ages correspond to 100–200 yr. Some remains of a relict stem, which was very probably partially fused with stem III, can still be observed toward east, see Fig. 5. Its collapse produced severe wounds to stem III. *Adansonia* species are tropical stem-succulent trees and also sapwood trees which do not form heartwood. Like other sapwood trees, baobabs exhibit self-healing ability deep within the stem [19,29]. The wounds of the interior xylem of stem III were repaired by secondary growth. These new growth/regrowth layers were dated for segments 13(a) and 13(b). The very irregular profile/relief of stem IV and some photographs of the canopy shape (Fig. 6) suggest that another stem collapsed sometime in the past in the vicinity of stem IV and damaged it. The radiocarbon age of segment 14(a) suggests that we also dated new growth/regrowth which repaired the wounds of stem IV.

### Number and ages of stems

Our long term research, based mainly on AMS radiocarbon dating, revealed that all large African baobabs, with a circumference over 16 m (corresponding to a conventional diametre larger than 5 m), are multi-stemmed. Even if more than 75% of young baobabs are single-stemmed, the vast majority of large and old baobabs are multi-stemmed. As baobabs age, single-stemmed specimens usually become multi-stemmed due to the baobabs’ capacity to generate new stems periodically, such as other tree species produce branches. This phenomenon occurs naturally, when new stems shoot from the roots or emerge from fallen stems. Over time, the new stems may fuse with older stem(s) or among them.

The structure of old baobabs can be established by using radiocarbon dates of samples collected from the inner cavity and from different areas of the multi-stemmed trunk. In addition, one can perform a visual inspection of the trunk, of the canopy shape and of the area where the trunk forks, in order to identify stems and possible fusion or separation/dividing lines.

In the case of Lebombo baobab, the very different ages of the oldest segments of the three cavity samples (1, 2 and 5) indicate that they originate from three different stems (I, II and V). The two short and young external samples, which consist of regrowth layers (13 and 14), originate from two other stems (III and IV), that are located beween stems II and V and are very obvious. The five extant stems, labelled I, II, III, IV and V, are displayed in Fig. 3. These five fused stems build a closed ring-shaped structure with a false inner cavity.

There are some indices that the Lebombo Eco Trail baobab was several centuries ago considerably larger than today. The remains of a relict stem, which was adjacent to stem III and external to the ring, can still be observed (Fig. 5). We also mentioned that in the past another stem existed, which was also external to the ring and adjacent to stem IV.

The calibrated ages of the oldest segments of the internal samples 1, 2 and 5, which were 995 ± 25, 1355 ± 15 and 835 ± 30 yr, can be used for determining the ages of stems I, II and V. According to the sample lenghts, we stated that the deepest part of the internal samples corresponds to a hollow area around the point of maximum age in the sampling direction. Our research suggests that typically ca. ± 50–100 yr around the points of maximum ages are missing in old baobabs. Therefore, in a conservative estimate, one can add ca. 50 yr to the age of the oldest segment for determining the age of the sampled stem. Consequently, the ages of stems I, II and V can be estimated to 1050, 1400 and 900 yr. The oldest stem II is the starting area of the ring, which has formed and closed progressively clockwise (II → I) and anti-clockwise (II → III → IV →V). One can state that stems III and IV have age values between the ages of stems II and V. The difference between the estimated ages of the oldest stem (II) and youngest stem (V) is close to the time which was needed for building the complete ring. On the other hand, stem III has a pronounced convex shape toward the exterior; this shows that it had to conform to the shape of the relict stem, which started growing several centuries earlier (Fig. 5).

The age of the oldest remaining part of the Lebombo Eco Trail baobab is that of stem III, *i*.*e*., 1400 ± 50 yr, which started growing toward ad 600. The closed ring was completed around 800–900 yr ago. By these values, the Lebombo tree becomes one of the oldest known African baobabs [21].

Nevertheless, one should mention that the point of maximum age in the sampling direction for cavity samples is typically younger than the true age of the stem, which corresponds to the age of its pith. This is due to the fact that even if we found some ring-shaped structures which are still developing or which remained incomplete, the successive emergence/sprouting of new stems and their growth in order to build the ring is still insufficiently elucidated.

### False cavities

The investigation of the Lebombo Eco Trail baobab, performed in 2010–2012, identified for the first time a new architecture of the African baobab: trees with false inner cavities. In the period 2010–2014, we visited and measured over 50 African baobabs with false cavities inside closed ring-shaped structures, out of which we dated 20 specimens by AMS radiocarbon. All dated trees showed the same major anomaly as previously found for the Lebombo baobab, namely the age sequence of samples collected from inner cavities exhibits a continuous increase from the cavity walls up to a certain distance into the wood, which corresponds to an area of maximum age. All these false cavities are natural empty spaces, which are located between several fused stems disposed in a closed ring-shaped structure. Such as the ring-shaped structures, the false cavities close progressively over time. The number of stems which build the ring is between three and eight. The thickness of the false cavity walls is usually between 1.5–2.5 m. The oldest part/point of maximum age of the fused stems is located between the false cavity walls and the exterior of each stem, always closer to the cavity, in an area which is accessible to the increment borer.

In large normal cavities, which occur by wood removal, the pith is located inside the cavity; therefore, the age sequence of samples collected from normal cavities decreases continuously toward the exterior. There are, however, other major differences between normal and false cavities, beside the sense in which the age sequence of the cavity samples varies.

Normal cavities usually have irregular shapes and are not very tall (1.0–2.7 m). By contrast, false cavities are larger and taller (3.0–8.3 m), have quasi-regular shapes and their lower part is located always at ground level. The first noticeable difference between false and normal cavities is the presence or absence of the bark inside the cavity. On the other hand, normal cavities become larger over time due to continuous decay, while false cavities become smaller because of stem growth.

According to our research, false cavities are much more frequently found in old baobabs than large normal cavities. AMS radiocarbon dating results indicate that the majority of the largest and oldest African baobab specimens from all-around the world exhibit closed ring-shaped structures with false cavities.

The circumference (cbh) of the dated baobabs with false cavities varies between 12.60 m (baobab of Gandiol, Thiès, Senegal) and 35.10 m (Holboom, Nyae Nyae Conservancy, Namibia). The age values of these specimens also spread over a wide range, between 300 yr (baobab of Gandiol) and 1750 yr (Holboom).

## Conclusions

This research reports the results of the radiocarbon investigation of the Lebombo Eco Trail baobab, a very large specimen from Mozambique. A number of three wood samples were collected from the inner cavity and other four samples from the outer part/exterior of the tree. AMS radiocarbon dating results demonstrate that, for all seven samples, the age values show a continuous increase with the distance/depth into the wood. For samples collected from the cavity walls, the increase of age values with the distance into the wood (up to a point of maximum age) represents a major anomaly. The only reasonable explanation for this finding is that such inner cavities are only natural empty spaces between several fused stems disposed in a closed ring-shaped structure. We named them false cavities. Several significant differences between normal cavities, which are formed by wood removal, and false cavities, which are only empty spaces between fused stems that were never filled with wood, are described.

Subsequently, we found and dated many other African baobabs with false inner cavities. We also found that this new baobab architecture, *i*.*e*., the closed ring-shaped structure with false inner cavity, enables baobabs to reach large sizes and old ages.

The radiocarbon date of the oldest sample segment was found to be 1425 ± 24 bp, which corresponds to a calibrated age of 1355 ± 15 yr. This result indicates that the oldest part of the Lebombo Eco Trail baobab has an age of at least 1400 ± 50 yr. The dating results also show that the Lebombo baobab consists of five fused stems, with different ages between 900 and 1400 yr; these stems build the complete ring. The ring and the false cavity closed around 800–900 yr ago. The investigation and dating results also show that two additional external stems, which were adjacent to the five-stemmed ring, collapsed and died around 200 yr ago.

We also found a second dating anomaly, namely that the innermost rings of the cavity samples, which are adjacent to the cavity bark, were found to be several hundred years old rather than being very young, as expected. The results demonstrate that the stems of the Lebombo baobab stopped completely growing toward the false cavity over the past 500 yr, while they continued growing at an almost constant rate toward the outer part.
